# New insights into the helical structure of 30-nm chromatin fibers

**DOI:** 10.1007/s13238-014-0080-x

**Published:** 2014-06-28

**Authors:** Ping Chen, Ping Zhu, Guohong Li

**Affiliations:** National Laboratory of Biomacromolecules, Institute of Biophysics, Chinese Academy of Sciences, Beijing, 100101 China

In eukaryotic cells, genomic DNA is highly packaged into chromatin to fit inside the nucleus. The accessibility of DNA is dependent on the packing density of chromatin fibers, which plays a critical role in transcriptional regulation and all other DNA-related biological processes. Understanding the structure of chromatin is key to illuminating the functions and molecular mechanisms of chromatin dynamics in epigenetic regulation. The structure of nucleosome—the basic unit of chromatin—has been defined by crystal X-ray studies at high resolution; it consists of 147 base pairs (bp) of DNA wrapping around an octamer of histones (two copies each of H2A, H2B, H3 and H4) approximately 1.7 times (Luger et al., 1997). Despite considerable efforts during the last three decades, however, how the nucleosomes interact with each other in a “beads-on-a-string” nucleosomal array to form a condensed 30-nm chromatin fiber—typically regarded as the secondary structure of chromatin—still remains controversial (Robinson and Rhodes, 2006). Based on the studies of native 30-nm fibers in nuclei or isolated from nuclei by electron microscopy, small angle X-ray/neutron scattering, optical dichroism and analytical centrifugation (Finch and Klug, 1976; Gerchman and Ramakrishnan, 1987; Ghirlando and Felsenfeld, 2008; Langmore and Paulson, 1983; Widom and Klug, 1985), two basic classes of structural models had been proposed in which nucleosomes are either arranged linearly in a one-start solenoid-type helix with bent linker DNA or zigzag back and forth in a two-start stack of nucleosomes connected by a relatively straight DNA linker (Widom and Klug, 1985; Williams et al., 1986; Woodcock et al., 1984). However, it is very difficult to resolve nucleosomes and linker DNA to trace the paths of nucleosomal arrays under conditions in nuclei or in isolated native chromatins in these early studies; in addition, the heterogeneous properties of nucleosomes in native chromatin with different DNA sequences/linker lengths and different histone compositions/modifications make it difficult to define the detailed structure of chromatin fibers. The problem has been partially addressed by reconstituting chromatin fibers *in vitro* on regular tandem repeats of unique nucleosome-positioning DNA sequences with purified histone proteins (Dorigo et al., 2004), which greatly improved the reproducibility and uniformity for structural analysis and allowed for a dissection of the contribution of individual factors, such as different NRLs, linker histones and histone variants/modifications. Using this system, the 3D cryo-EM structures of 30-nm chromatin fibers reconstituted *in vitro* from arrays of 12 nucleosomes in the presence of linker histone H1 have been recently determined at resolution of about 11 Å, which clearly show a histone H1-dependent left-handed twist of the repeating tetra-nucleosomal structural units (Song et al., 2014). The structures constitute the largest fragments of chromatin solved at this resolution which is high enough to allow clearly defining the spatial location of all individual nucleosomes and tracing the path of linker DNA, and provide new insights into the helical structure of 30-nm chromatin fibers.

**Figure 1:**
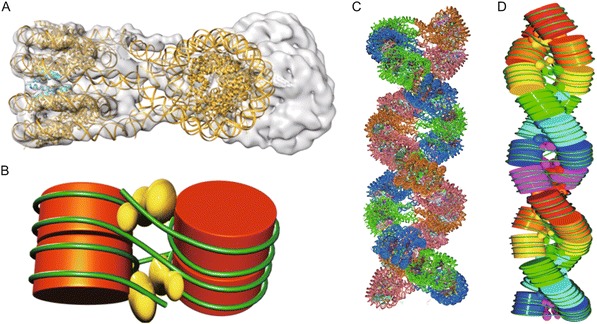
**New structural insight into the 30-nm chromatin fibers**. (A) The segmented cryo-EM density map (grey) for the tetra-nucleosomal unit in the 30-nm chromatin fibers reconstituted on 12 × 187 bp DNA, with a comparison to the X-ray structure (PDB ID: 1ZBB, yellow) of the tetra-nucleosome (Schalch et al., 2005). (B) A schematic representation of the cryo-EM structure for the tetra-nucleosomal unit as shown in A. (C) A pseudo-atomic model (structure of H1 is not included) built from the cryo-EM structure of the dodeca-nucleosomal 30-nm fiber. (D) A schematic representation of the cryo-EM structure for the 30-nm chromatin fibers

The 3D cryo-EM structures of 12-nucleosomal 30-nm fibers reconstituted with two different nucleosome repeat lengths (NRLs), 177-bp and 187-bp 601 DNA sequence, are resolved with very similar overall architecture which comprises three tetra-nucleosomal structural units. An increase of 10-bp NRL does not affect the overall structure of chromatin fiber, but changes the fiber dimension from approximately Φ27.2 × 28.7 nm for 177-bp NRL to approximately Φ29.9 × 27.0 nm for 187-bp NRL, which is consistent with the fundamental prediction of a basic zigzag two-start helix model. The four nucleosomes within the structural unit zigzag back and forth to form two stacks of two nucleosome cores connected by straight linker DNA, which appear very similar to the resolved X-ray structure of a tetra-nucleosome with the 167-bp NRL in the absence of linker histone (Schalch et al., 2005) (Fig. 1A and 1B). The structures for each stack of two nucleosomes fit very well by docking the X-ray atomic structure of the 167-bp NRL into the EM density map of the structural units with 187-bp and 177-bp NRLs. The results indicate that the presence of H1 and the length change of linker DNA do not affect the interactions within the nucleosome stack, but affect the separation and rotation between the two stacks. The two stacks are separated by 146.1 Å with 71.3° left handed rotation for 167-bp NRL in the X-ray structure, compared to 167 Å with 63.7° left handed rotation for the 177-bp NRL and 196 Å with 54.5° left handed rotation for the 187-bp NRL in the cryo-EM structures.

The tetra-nucleosomal structural units are twisted left handed against each other to form the final helical structure of 30-nm fiber. The H1-H1 interaction and the interactions of the nucleosomal interfaces between tetra-nucleosomal structural units play central roles in the formation of helical twist in 30-nm fiber. The linker histone has long been considered to be essential for the formation 30-nm chromatin fiber (Allan et al., 1980; Bates and Thomas, 1981; Thoma et al., 1979; Thomas, 1999). However, the precise role of linker histone in the 30-nm chromatin fiber remains to be structurally determined. The resolved cryo-EM structure, for the first time, clearly visualizes and addresses the location and the precise role of H1 in the formation of 30-nm chromatin fiber. The H1, exhibiting a proper 1:1 stoichiometric association with the nucleosome cores in the 30-nm fiber, directly interacts with both the dyad and the entering and exiting DNAs in a three-contact mode. In addition, the H1 locates asymmetrically in each nucleosome core with its bulk of globular domain pointed outside the structural units, which allows the self-association of H1 between tetra-nucleosomal units and imparts an additional twist between each structural unit. Furthermore, the inter-unit interactions between the positively charged residue of the H4 N-terminal tail (Arginine 23) and the acidic patch of the H2A-H2B dimer on the face of the opposed nucleosome also account for the twist between the tetra-nucleosomal units. In comparison to the closely stacked nucleosomes within the tetra-nucleosomal structural unit, the apparently gaps formed between tetra-nucleosomal units may provide a platform for histone modifications or other architectural proteins to modulate the inter-nucleosomal surface interactions in the regulation of high-order chromatin structures.

How nucleosomal arrays fold into 30-nm chromatin fibers has been remained as one of the fundamental questions in molecular biology for over three decades. The cryo-EM structure of 30-nm fiber at high resolution provides a new paradigm for further investigation into the epigenetic regulation of chromatin structures. The double helical model for the 30-nm chromatin fiber in the presence of H1 could notably advance the understanding of how DNA and histones compact into higher-order chromatin fibers (Fig. 1C and 1D). It is also worth noting the physiological relevance of the 30-nm chromatin fiber. Although the existence of 30-nm fibers *in vivo* remains to be elucidated (Maeshima et al., 2010), fibers assembled under physiological conditions *in vitro* can be used to define the molecular mechanisms of chromatin dynamics. It is also of great interest to note that the double helical structure of DNA (Watson and Crick, 1953) ensures the inheritance of the genetic information through DNA replication. The double helical model for 30-nm chromatin fiber (Fig. 1C and 1D), which is built by a left-handed twisted repeating tetra-nucleosomal unit mainly via histone H1-H1 interaction, could potentially provide a structural basis for the inheritance of epigenetic information, such as histone modifications. Recent findings that the proximity of nucleosomes (Yuan et al., 2012), the presence of histone H1 (Martin et al., 2006) and pre-existence of H3K27me3 (Margueron et al., 2009) can strongly stimulate the polycomb repressive complex 2 (PRC2) activity to regulate H3 lysine 27 methylation, suggest that the higher-order chromatin structures may play important roles in the propagation of epigenetic information (Li and Reinberg, 2011; Margueron and Reinberg, 2010). In addition, many epigenetic factors, including DNA methylation, histone modifications, histone variants and the binding of non-histone architectural proteins have been shown to be crucial in regulating the dynamics of chromatin structures. It will be of particular interest to further investigate how these factors regulate the folding of chromatin fiber, which is essential for understanding the structural principles of chromatin dynamics and its correlation with gene regulation.
